# Learning “How to Learn”: Super Declarative Motor Learning Is Impaired in Parkinson's Disease

**DOI:** 10.1155/2017/3162087

**Published:** 2017-07-30

**Authors:** Lucio Marinelli, Carlo Trompetto, Stefania Canneva, Laura Mori, Flavio Nobili, Francesco Fattapposta, Antonio Currà, Giovanni Abbruzzese, Maria Felice Ghilardi

**Affiliations:** ^1^Department of Neuroscience, Rehabilitation, Ophthalmology, Genetics, Maternal and Child Health, University of Genova, Genova, Italy; ^2^Ospedale Policlinico San Martino, Genova, Italy; ^3^Neurology Unit, Policlinico Umberto I, Department of Neurology and Psichiatry, Sapienza University of Rome, Rome, Italy; ^4^Academic Neurology Unit, A. Fiorini Hospital, Terracina (LT), Department of Medical-Surgical Sciences and Biotechnologies, Sapienza University of Rome, Polo Pontino, Italy; ^5^Department of Physiology, Pharmacology & Neuroscience, CUNY School of Medicine, New York, NY, USA

## Abstract

Learning new information is crucial in daily activities and occurs continuously during a subject's lifetime. Retention of learned material is required for later recall and reuse, although learning capacity is limited and interference between consecutively learned information may occur. Learning processes are impaired in Parkinson's disease (PD); however, little is known about the processes related to retention and interference. The aim of this study is to investigate the retention and anterograde interference using a declarative sequence learning task in drug-naive patients in the disease's early stages. Eleven patients with PD and eleven age-matched controls learned a visuomotor sequence, SEQ1, during Day1; the following day, retention of SEQ1 was assessed and, immediately after, a new sequence of comparable complexity, SEQ2, was learned. The comparison of the learning rates of SEQ1 on Day1 and SEQ2 on Day2 assessed the anterograde interference of SEQ1 on SEQ2. We found that SEQ1 performance improved in both patients and controls on Day2. Surprisingly, controls learned SEQ2 better than SEQ1, suggesting the absence of anterograde interference and the occurrence of learning optimization, a process that we defined as “learning how to learn.” Patients with PD lacked such improvement, suggesting defective performance optimization processes.

## 1. Introduction

Efficient learning of motor skills, which occurs daily in our lives, involves cognitive processes at least in the initial stages of learning [1]. For example, in the early phase, attentional mechanisms at different levels are needed, while later on, such processes become less important as automatization gradually occurs. An important factor limiting the ability to learn new information or retain and retrieve previously learned material is the interference caused by learning other information. Specifically, anterograde interference occurs when the learning task A interferes with or delays the successive learning of task B; retrograde interference, instead, occurs when the successive learning of task B interferes with the successful performance of previously learned task A. Factors determining the occurrence and the degree of interference are the following: the time interval between two tasks (the longer the time interval the smaller the interference), the amount of practice for the specific task (the more the practice the smaller the interference), and the sharing of the neural bases [2]. The two types of interference are present for both declarative and procedural aspects of a motor task, although it seems that declarative aspects are more susceptible to anterograde interference while others are more susceptible to retrograde interference [3]. Notably, consolidation occurs when a skill is learned extensively and is no longer vulnerable to retrograde interference by a subsequent task [3, 4]. Undoubtedly, the retention of previously learned material is greater if performance saturation is achieved during training and, most importantly, if sleep occurs between training and testing. Indeed, it has been shown that in normal subjects, better retention of both declarative material and motor skills occurs after a period of posttraining sleep than after a similar period of wakefulness [1, 5].

PD is a disease characterized by a decrease of cortical plasticity and therefore by abnormal retention of learned material. In fact, few studies on retention of motor skills in patients with PD have reported impaired retention, despite intact initial learning [6–14]. Interference in PD has not been thoroughly explored: to date, there is only one study investigating anterograde interference in PD with a visuomotor transformation task. The results demonstrated a reduced effect of anterograde interference that was interpreted as the consequence of a decreased LTP-dependent plasticity induced by dopamine deficiency [15]. In the same study, both PD and controls reached a comparable initial learning of the visuomotor transformation. Similar to Leow et al., we used an arm-reaching task; however, instead of triggering implicit motor learning, our sequence learning task elicits declarative learning measured by the number of movements correctly anticipated toward targets of a repeating sequence. We studied anterograde interference induced by the first learned sequence to a different sequence that the subjects performed afterwards. We also evaluated how declarative learning of a sequence is retained after a night of sleep.

The aim of this exploratory study is to understand if early untreated PD patients are able to retain declarative motor memories and whether such consolidated task may exert anterograde interference on a subsequent declarative learning task.

## 2. Materials and Method

### 2.1. Subjects

Subjects were eleven drug-naive patients (9 men) newly diagnosed with idiopathic PD, as per the established guidelines [16]. The mean age was 64.8 years (±3.4 SD), Hoehn-Yahr stage was 1-2, and the UPDRS-III mean score was 15 ± 2. The most involved side was the right in four patients and the left in the remaining seven. Eleven age-matched normal controls (64.4 ± 1.8 years) were also recruited. Both patients and controls were all right handed based on the Edinburgh Handedness Inventory [17], and none had significant cognitive impairment, as confirmed by a neuropsychological assessment including the Mini-Mental State Examination (MMSE) where all patients scored more than 26. A written informed consent was obtained from all participants. This study has been carried out in accordance with the Code of Ethics of the World Medical Association (Declaration of Helsinki) for experiments involving humans. The study was approved by the local ethics committee.

### 2.2. Motor Task

The experimental setup has been described in detail in previous works [3, 18, 19]. Subjects performed two motor tasks. Briefly, in both tasks, they moved a cursor on a digitizing tablet with their right (dominant) hand. One of eight radially arranged targets displayed on a computer screen with a central starting point (distance of 4.8 cm) turned black in synchrony with a tone at fixed intervals of 1.5 s. Instructions were to make fast, accurate, and uncorrected movements out and back from the central starting point to the highlighted target, reversing sharply inside the target's circle within a set time window. A successful hit was indicated by the target turning gray. Subjects received a feedback about their performance at the end of each block.

In one task (RAN), the targets appeared in an unpredictable, random order and subjects were trained to reach the target in a time window of 500 ms from its appearance. This task was used to determine, for each subject, the minimum reaction time or floor reaction time and, thus, to compute the number of correct anticipatory movements in the sequence learning task (SEQ), as described below. In this task (SEQ), subjects were instructed to learn the order of a repeating 8-target sequence while reaching for the targets. In addition, subjects were required to start the movement in advance and hit the target in synchrony with its appearance when the target location was predicted (and thus learned). We used two sequences of equal complexity (SEQ1 and SEQ2). Each sequence was presented in blocks of 10 repetitions: every 2 blocks, the subjects were asked to verbally report the sequence order and a verbal declarative score (from 0–8) was acquired [18]. In addition, we computed the number of correctly anticipated movements (CAM) in each SEQ block. Correct anticipatory movements were defined as those with reaction time below the floor reaction time in RAN and directional error at peak velocity less than 22°.

**Figure 1:**
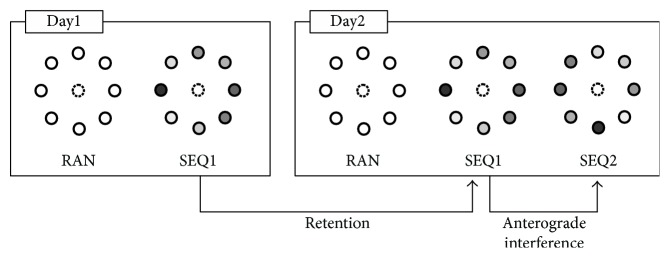
Experimental paradigm. Random multiple-choice reaction time tasks (RAN) preceded sequence learning tasks (SEQ1 and SEQ2) during two consecutive days (Day1 and Day2). Both SEQ1 and SEQ2 sequences had a comparable complexity. The comparison of learning indices (correctly anticipated movements (CAM)) of SEQ1 between Day1 and Day2 allowed the assessment of declarative learning retention; the comparison of SEQ1 at Day1 and SEQ2 at Day2 allowed the assessment of anterograde interference of repeated learning of SEQ1 over SEQ2. Shades of gray reflect the actual target sequence (lighter to darker).

All subjects were naive to the tasks and therefore were training in the days before the testing sessions. The data obtained from the training were not included in the analysis. As in previous works [20], regression analysis confirmed a significant correlation between correct anticipatory movements and declarative scores both in PD (*r* = 0.47, *p* = 0.005) and controls (*r* = 0.79, *p* < 0.0001). Therefore, the analyses are focused on correct anticipatory movements. Study design is illustrated in Figure 1().

The motor tasks were performed during two consecutive days (Day1 and Day2 sessions). On Day1, two blocks of RAN (80 movements each) tasks were followed by SEQ1. SEQ1 was presented in blocks of 80 movements, corresponding to 10 complete presentation of the 8-target sequence. As the goal was to have all the subjects learning the sequence at their best and reaching a plateau, the number of blocks was different for each subject ranging from two to six blocks. The following day, at the same time, subjects performed two blocks of RAN and four SEQ1 blocks with the same sequence of Day1. After the verbal report of SEQ1 order, subjects were informed that a different sequence was being presented in SEQ2 and two blocks of SEQ2 were performed followed by the verbal report of SEQ2 order.

### 2.3. Outcome Measures and Statistics

Declarative learning across movements during sequence learning was measured with CAM. Sequence retention index was computed by comparing the number of CAM during the first 160 movements of SEQ1 in Day1 and that in Day2. Anterograde interference was evaluated comparing the number of CAM of the first 160 movements of SEQ1 in Day1 and CAM of the first 160 movements of SEQ2 in Day2. Anterograde interference of SEQ1 on SEQ2 was reflected by a lower number of CAM in SEQ2 at Day2 than CAM in SEQ1 at Day1.

Comparisons between SEQ1 at Day1, SEQ1 at Day2, and SEQ2 were performed with a mixed-model ANOVA with GROUP (PD and controls) as main factor and SEQ (SEQ1 Day1, SEQ1 Day2, and SEQ2 Day2) as within factor (three time points). Each subject entered analysis considering the first 20 sequence repetitions (20 repetitions × 8 targets = 160 movements).

Bonferroni's test was used for post hoc comparisons. Differences were considered significant if *p* < 0.05, and variability was always measured as standard deviation.

## 3. Results

Both PD and controls became quickly familiar with the motor task and were able to perform straight out and back movements. On Day1, none of the PD patients was able to report the complete sequence order of the first two SEQ1 blocks (average declarative score ± SE: 3.3 ± 0.6) and only four controls reported the complete order (average 5.3 ± 0.9).

**Figure 2:**
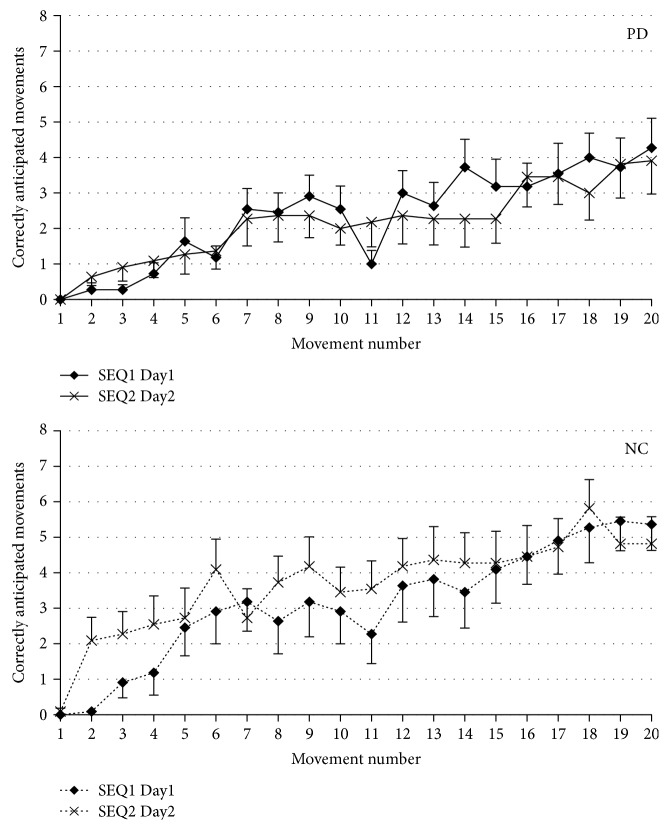
Time course of correctly anticipated movements (CAM) in PD patients (solid line) and normal controls (dotted line) during for SEQ1 at Day1 and SEQ2 at Day2. In PD, SEQ1 and SEQ2 are learned in a similar way, while in controls, SEQ2 is learned better, reflecting “learning how to learn.” Vertical bars report variability as standard error.

A mixed model ANOVA of CAM further indicated that patients had a reduced learning rate compared to controls (GROUP: *F*[1, 876] = 28.5, *p* < 0.0001), with a significant GROUP × SEQ interaction (*F*[1, 2] = 4.2, *p* = 0.016) and with a significant effect of SEQ (*F*[2, 876] = 182.9, *p* < 0.0001). These results indicate that two groups performed differently in some of the tests, with the PD group having worse performance in the three SEQ tests. To better investigate the significant GROUP × SEQ interaction, we analyzed the two groups separately. Post hoc analyses showed that performance was more successful for SEQ1 on Day2 than for SEQ1 on Day1 for both controls and PD (*p* < 0.0001), suggesting that some retention had occurred in both groups. Also, in both groups, SEQ1 performance on Day2 was more successful than SEQ2 in both groups (post hoc: *p* < 0.0001). More interestingly, we found a greater learning rate of SEQ2 compared to that of SEQ1 on Day1 in the control group (*p* = 0.0028) but not in PD patients (*p* = 0.3) (Figure 2). This finding indicates that, in control subjects, anterograde interference did not occur but the learning rate in SEQ2 was faster than that in SEQ1 the previous day, despite the same degree of complexity of the two sequences. In fact, in case of anterograde interference, the newly presented SEQ2 should have been learned less efficiently than SEQ1 at its first presentation on Day1. The significant improvement in the control group of SEQ2 compared to that of SEQ1 on Day1 suggests the occurrence of an improvement in the general mechanisms involved in sequence learning that is an improvement in “learning how to learn.”

**Figure 3:**
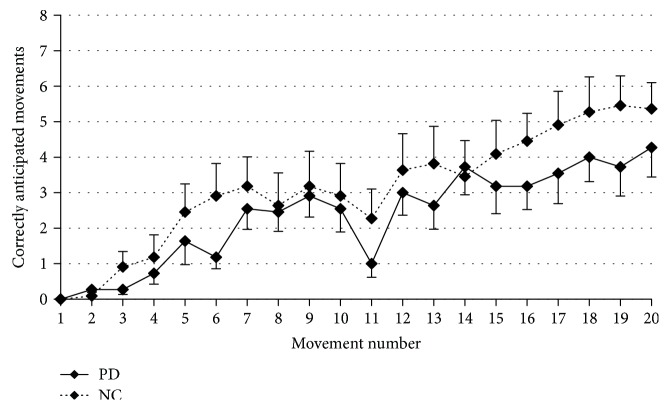
Direct comparison of the time courses of CAM in PD (solid line) and controls (dotted line) during SEQ1 at Day1, reflecting a reduced learning process in PD patients. Vertical bars report variability as standard error.

**Figure 4:**
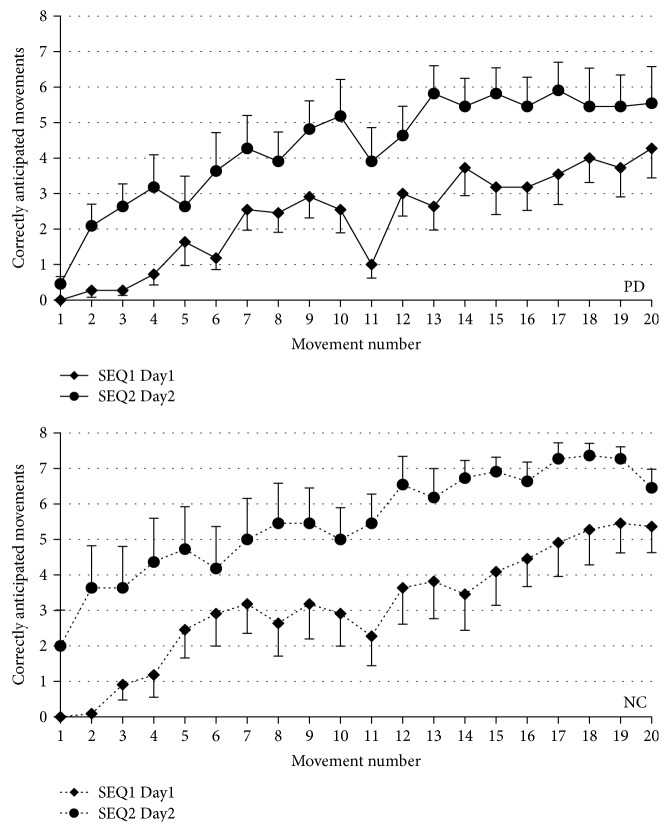
Time course of CAM in PD (solid line) and controls (dotted line) between SEQ1 at Day1 and SEQ1 at Day2, reflecting sequence retention after a night of sleep. Both groups undergo retention, even if overall, the number of CAM is higher in controls. Vertical bars report variability as standard error.

Defective declarative sequence learning in PD was confirmed by a factorial ANOVA showing that PD learned SEQ1 worse than the controls (GROUP: *F*[1, 438] = 9.5, *p* = 0.002) (Figure 3). When tested the day after, the performance at SEQ1 was better than that on Day1, although retention of SEQ1 was significantly worse in PD than in controls, as shown by a mixed-model ANOVA comparing SEQ1 at Day1 and SEQ1 at Day2 (GROUP: *F*[1, 438] = 16.8, *p* < 0.0001; SEQ: *F*[1, 438] = 335.8, *p* < 0.0001) (Figure 4). To compare retention between the two groups despite this difference in learning levels, we considered the interaction GROUP × SEQ. This interaction did not reach significant level (*F*[1, 438] = 3.3, *p* = 0.07), suggesting similar retention levels in the two groups.

## 4. Discussion

This study aims to understand for the first time whether patients with PD are able to retain declarative motor memories after a night of sleep and whether the learned material determines anterograde interference on a subsequent declarative learning task.

In order to prevent the confounding effect of dopaminergic treatment as well as motor and nonmotor complication that may occur in the later stages of the disease, we recruited only drug-naive patients in the early stages of the disease. Compared to age-matched normal controls, our patient population showed a reduced learning rate of an 8-target sequence SEQ1, while its retention after a night of sleep was similar in the patients and the controls. Anterograde interference of SEQ1 over SEQ2 was not evident, as the learning rates of SEQ2 and SEQ1 were similar. Unexpectedly, not only anterograde interference was lacking but also normal subjects learned SEQ2 better than SEQ1. This sort of “learning how to learn” effect was missing in PD patients.

### 4.1. Declarative Motor Learning Retention

Our data are in line with previous studies employing an experimental paradigm similar to ours, demonstrating impaired declarative motor sequence learning in PD [10, 18, 21–23]. We avoid discussing studies performed with the serial reaction time task, because of the mixed declarative/procedural learning triggered by the task, as explained in the introduction [24]. When learning a visuomotor transformation task lacking declarative components, PD patients behave like normal controls, probably because implicit learning does not impinge on working memory and attentional resources that can already be affected in the early stages of the disease [9, 25].

When learned material needs to be stored in memory, cortical networks are involved in triggering retention, where the role of dopaminergic innervation is crucial [26]. As we previously demonstrated, retention of procedural learning is impaired in PD [9] and evidences point to the important role of right posterior parietal area [12, 27, 28].

Consolidation of declarative learning in PD is underexplored. Using a nonmotor episodic memory task, not drug-naive PD patients showed impaired retention strongly related to dopamine on or off conditions [26]. Conversely, drug-naive PD patients tested with a serial reaction time task showed intact consolidation [29]. Our data point toward an effective retention of declarative motor learning in drug-naive early PD, despite impaired learning. It is possible that the upregulation of endogenous dopaminergic innervation of anterior cortical areas could play a role in preserving retention of declarative memories [30, 31]. We believe that such finding should be considered preliminary because of the marginal significance of the statistical analysis and the limited number of subjects, thus encouraging specifically designed studies in drug-naive patients using experimental paradigms that enhance declarative components of motor learning.

An effect of disease lateralization, hand dominance, and the body side performing the motor task were taken into account in some works [29, 31]. In our study, all subjects were right handed and all performed the task with the right hand; 4 patients had the right side more involved by the disease, and 7 the left side. This study was not designed to analyze the effect of lateralization; however, impaired motor skills associated to an increased reaction time or reduced movement speed in relation to the use of the nondominant hand or that more affected by PD are not likely to affect the number of CAM. In fact, minimal reaction time was previously calculated individually based on a multiple-choice reaction time task and used as a threshold to detect anticipatory movements. Also, movement speed does not influence the number of CAM since all subjects were able to complete the movements before the appearance of the following target.

### 4.2. Anterograde Interference and “Learning How to Learn” Effect

In normal subjects, interference between consecutive motor tasks occurs frequently and has been related to different mechanisms involving neural plasticity and connectivity between brain areas. During motor learning, long-term potentiation (LTP) occurs in the primary motor cortex (M1). Consequent learning tasks progressively degrade because less LTP is available for the learning processes, actually determining anterograde interference. Since LTP is restored over time, interference decreases as time interval between learning processes increases [32]. Anodal transcranial direct current stimulation of M1 is capable of increasing consolidation of a previously learned sequence by inducing LTP processes. Consequently, a new sequence learned after the stimulation induced less retrograde interference on the first sequence, while the new sequence is learned less efficiently because of both the increased anterograde interference exerted by the first sequence and the reduced availability of LTP resources. This mechanism was described as “occlusion” [2].

In PD, an impairment of LTP-related processes was demonstrated [33–37], providing the basis for both reduced retention and reduced interference. In the present study, we did not observe anterograde interference in PD since SEQ2 was not learned worse than SEQ1 despite that both sequences had the same complexity and SEQ1 was already learned and relearned after 24 hours, therefore expecting even higher anterograde interference. In normal controls, we also expected anterograde interference; however, not only it was absent but we also observed the opposite: SEQ2 was learned better than SEQ1. The lack of anterograde interference also in controls cannot explain these results, since the simple lack of anterograde interference would have caused SEQ1 and SEQ2 to be learned to the same extent (as occurred in the sole PD group). We therefore hypothesize that another phenomenon intervened, causing the control subject to take advantage of the very process of learning SEQ1 to improve SEQ2 learning. To be noticed, all subjects were naive to the task, so they never performed sequence learning tasks before. If subjects were trained before, we probably would not be able to observe such increased learning effect.

This improved learning that we call “learning how to learn” is therefore present only in control subjects but not in PD. We could find a previous description of such phenomenon in another study where using a procedural finger-tapping motor sequencing task controls but not medicated PD performed better in the second half of the postsleep retest session compared to their posttraining learning [13]. This “learning how to learn” effect can be interpreted as a “super declarative” learning selectively impaired in PD.

## 5. Conclusions

The points of strength of this study are that only drug-naive early PD patients have been recruited, thus avoiding the confounding effect of dopaminergic treatment. Moreover, differently from other studies adopting serial reaction time tasks, the motor task we adopted is highly specific for declarative learning. On the other hand, the limitations of this study are represented by the limited number of subjects, partly due to the difficulty in finding newly diagnosed drug-naive PD patients, the lack of specific assessments to demonstrate the role of sleep on declarative learning retention, and also the inability to make a direct comparison of retention between PD and controls.

In conclusion, this exploratory study for the first time suggests that consolidation of declarative learning in PD is not significantly impaired and that in the early phases of declarative sequence learning, anterograde interference is replaced by a progressive improvement of sequence acquisition in normal subjects but not in PD patients.
